# Progress in General Surgery

**Published:** 1901-07-20

**Authors:** 


### PROGRESS IN GENERAL SURGERY.

(*Continued from page* 181.)

**Fractures and Dislocations** (*continued*), **Upper Extremity.**—A rare instance of fracture of the clavicle resulting in rupture of the suprascapular artery is recorded by Dr. H. T. Miller.[14] A married woman, aged 45, fell in an epileptic attack four weeks before admission, and upon regaining consciousness discovered a swelling over the middle of the right clavicle. This swelling grew rapidly with considerable pain. A large pulsating tumour was found extending from the omohyoid muscle to the fifth rib and from the suprasternal fossa to the axilla. Two incisions were made dividing the sterno-mastoid sterno-hyoid and sterno-thyroid muscles and the subclavian artery exposed. The finger was inserted between the artery and vein into the tumour, which was largely composed of disorganised blood-clots. A portion of the clavicle was removed, and was found to have been fractured and partly necrosed, probably from pressure. A drainage tube was inserted, but although the wound was irrigated with antiseptic solution and all loose blood-clots removed, hæmorrhage still continued. Another incision was made over the clavicle with a view to cut down upon the suprascapular artery, but the hæmorrhage ceased and the ruptured vessel was not found, though 3 inches of the clavicle was resected. A pointed spicule of the clavicle had evidently been the cause of the rupture of the artery. Unfortunately the wounds became foul smelling, the tumour assumed its original size, and the patient died at the end of two months in a greatly emaciated condition. In describing a case of the very common dislocation upwards of the outer end of the clavicle, James Cantlie[15] states that the diagnosis of this condition is not difficult, the reduction of the dislocation is usually quite easy, but the subsequent attempt to keep the bones of the articulation in their place is always disappointing. This is accounted for by the direction of the obliquity of the articular surfaces, the clavicle tending to slip up on the acromion. The articulation depends for its strength upon the superior acromio-clavicular ligament, and as this is torn during displacement, the rational treatment would appear to be to regenerate the ligament. This cannot be done by pads or by rest, but by movement, so that the irritation kept up may induce an inflammatory thickening of the ligaments around the joint. He recommends keeping the shoulders back, and the limb of the affected side at rest in a sling for eight or ten days, and after that interval to allow of movement with the idea of causing thickening of the ligaments above the articulation. Fracture of the acromion process is at times mistaken for separation of the acromial epiphysis, but the diagnosis after a skiagram has been taken must be corrected by careful anatomical inspection, and by measurements. Mouchet[16] points out the fact that fractures of the neck of the radius as shown by the Roentgen rays are much more frequent than has been supposed. They occur in children between nine and twelve years. His observations are based upon eleven cases which came under his care within a few years. They are not as often associated with other fractures about the elbow as has been supposed. The attitude of the patient is almost pathognomonic. The elbow is flexed with swelling of the forearm muscles and pain on pressure in the region of the radial head and neck. The pain is increased by attempts at forcible supination. Abnormal mobility and crepitus are frequently absent, while active supination is always impossible. The treatment by massage and passive motion is simple, but reduction is often impossible. If therefore there remains functional loss, the head of the radius shall be cut down on and removed. A case of ischæmic paralysis in an adult aged 30 is recorded by Mr. Edmund Owen.[17] He believes that another factor besides pressure on the vessels and laceration and the subsequent contraction of the muscles is to be found in neuritis of the motor nerves of the part resulting from excessive pressure of the splint. It is probable that in such instances both causes act. The pathology of the case recorded was probably this, that the tight and long-continued compression of the forearm kept the muscles completely starved of blood, and so injured them that a myositis supervened, which, in its turn, was followed by an intractible fibroid degeneration of the tissue with permanent contracture. Dr. J. G. Sheldon[18] narrates a rare instance of dorsal dislocation of the trapezoid, unaccompanied with fracture or dislocation of other bones. In the only other case recorded by G. W. Gay the deformity could not be reduced at all, but in Sheldon's it was partially reduced. In both the deformity remained, although the patients could use the wrist as well as ever. Dr. Sheldon thought that in both there was a congenital weakness or absence of the ligaments, or a maldevelopment of the carpal bones, or a combination of these conditions which rendered it possible for an uncomplicated dislocation of the trapezoid to be produced. Mr. H. L. Barnard[19] has recently successfully employed a method of reduction of dorsal dislocation of the first phalanx of the little finger by the dorsal incision recommended by Desault, Hulke, Farabœuf, and Hutchinson junior for the more common dislocation of the same joint of the thumb. He entered a narrow-bladed knife on the dorsal surface of the joint to the ulnar side of the extensor tendon and carried the back of the blade along the articular surface of the base of the phalanx until the point of the knife impinged against the back of the metacarpal bone. He then cut firmly upwards for a distance of about one third of an inch, dividing the glenoid ligament upon the back of the metacarpal head. The ligament in the case recorded was distinctly felt to give when the section was complete. The metacarpal bone was then flexed upon the palm of the hand to relax the flexor brevis minimi digiti and the first phalanx was hyperextended and at the same time carried bodily forwards, when it immediately slipped into its place. Recovery of the patient was uneventful.

**Lower Extremity.**—Two cases of delayed union after simple fracture of the shaft of the femur, are related by Dr. W. H. Shipps.[20] The one was a young lady aged 17, the other a young man aged 26. From a study of these he concluded that delayed union is possible, notwithstanding the conditions for repair are apparently every way favourable; that consolidation is readily effected by systematic employment of massage and the use of the hypophosphites. He advises the employment of massage as early as the fourth week, both for the purpose of facilitating consolidation by improving the nutrition of the parts and to prevent knee stiffness or ankylosis, a not infrequent accompaniment of such injuries. The early use of the hypophosphites of lime and soda he advocates in all cases of fracture when there exists doubt of prompt bony union. In a lengthy paper on the structure and fracture of the patella, E. M. Corner[21] draws attention to a matter of common knowledge, viz., that the patella is not infrequently refractured. Bégonin and Anderodius have quoted a number of cases by Ham, Brummen, and Lavise, besides their own, to show that refracture is commoner after treatment by massage than otherwise. This method usually yields short and strong fibrous union. Powers states that a refracture is less frequent after wiring than after fibrous union. According to Corner's statistics from St. Thomas's Hospital, the frequency of refracture was as follows:—Four times fractured in two cases; three times fractured in three cases; two times fractured in 28 cases; one fracture of the other patella, three cases. From a study of the mechanics of the region he argues that oblique fractures of the patella might be considered to be far fewer than would be expected. Powers says that the usual direction of the obliquity is from above downwards, outwards, and inwards—*i.e.*, when the knee is fully flexed. From the St. Thomas's sources, Corner is inclined to agree with him. The insertion of the vastus internus into the upper and inner quadrant of the patella gives a slightly inward pull to the line of action of the resultant of the quadriceps, which will naturally lead to an obliquity of the fracture line. This will be from above downwards and without inwards, agreeing with what Powers has said. The total number of fractures admitted into St. Thomas's Hospital during 10 years was 272, and 254 were due to indirect violence and 18 due to direct violence. This gives the practical proportion of 14 to 1. Corner's table also shows that fractures of the patella are less common in females than males in a ratio greater than is the case with other fractures of the lower limb. They are really relatively most common in males for the patella and in females for the femur. After a criticism of the various methods of non-operative or mechanical and operative treatment of fractured patella, W. B. Trimble[22] concludes that an operation should not be attempted after the fiftieth year. Other contra-indications are diabetes and albuminuria; syphilis, gout, alcoholism, and tuberculosis also call for serious deliberation. However, taking everything into consideration, this writer believes that in most cases when rigid asepsis can be observed, the wire suture method is decidedly the best form of treatment. E. M. Cox[23] thinks that the entire non-absorbability of the wire is a decided objection to its use, and in spite of the fact that many patients are now carrying the wire without any evidences of irritation, we cannot possibly be certain that all will continue to do so indefinitely, and until we are certain that no patient will need to have the sutures removed and possibly a scraping operation done for localised caries, we must admit that the principle of using silver wire is defective, though the practice is apparently attended with success. Absorbable sutures are available to which these objections are not applicable. However, movements violent enough to break a good catgut suture should not be allowed after the suturing has been done. Cox quotes three cases which illustrate the results after the use of catgut sutures, in the first of which the material was prepared by the ether-alcohol-bichloride of mercury method, and in others by the formalin method. The results showed that the absorbable suture can be used successfully for holding together the fragments of a broken patella. A recent article by H. Zuppinger[24] gives 22 clinical observations on the production of spiral fracture of the bones of the leg, together with conclusions as to the reproduction of this variety of fracture experimentally by torsion. External rotation of the foot by force acting on the anterior half of the foot causes a spiral fracture in the lower half of the tibia, terminating posteriorily and a fracture of the fibula at a higher level. The direction of the spiral is right-sided on the left foot and vice versâ for the right. Inward rotation of the foot by forces acting on the anterior half of the foot causes a spiral fracture of the fibula at a lower level, eventually with fracture of the internal malleolus. Henry R. Wharton[25] relates an unusual case of fracture of the fibula with marked displacement of the lower fragment treated by operative interference. It occurred in a patient aged 23 years, who, while riding a bicycle, was struck by the fender of a trolley-car, and sustained a fracture of the left fibula about 2½ inches above its lower end, and a fracture of the internal malleolus with great contusion of the soft parts. Ten days after the injury, when the swelling had somewhat subsided, it was found that the upper end of the lower fragment projected upwards, well above the lower end of the upper fragment, and although it was possible to partially reduce the deformity by manipulation, as soon as the reducing force was removed the deformity recurred. Under ether, an incision was made over the seat of fracture. Upon exposing it, a strip of muscular tissue of the peroneus tertius was found between the ends of the fragments. This was displaced, the ends of the fragments drilled, and a heavy silver-wire suture was introduced and secured, holding the fragments in good position. The wound was closed without drainage, and the limb put up in moulded binder's board splints. The superficial sutures were removed on the tenth day, as the wound was healed. A plaster of Paris bandage was applied for six weeks and firm union resulted. In a large number of fractures of the fibula which have come under Wharton's observation, he had never before seen a similar deformity, and from the conditions found to exist at the seat of fracture it was not possible that the deformity could have been remedied, or satisfactory union could have occurred other than by operative interference.

[14] New York Med. Rec., Dec. 22, 1900, p. 973. [15] Clin. Journ. Nov. 1900, p. 39. [16] Ann. Journ. Med. Science, Oct. 1900, p. 481. [17] Lancet, Feb. 2, 1901, p. 321. [18] Amer. Journ. of Med. Sciences, Jan. 1901, p. 85. [19] Lancet, July 12, 1901, p. 81. [20] Med. Rec., New York, Sept. 22, 1900, p. 456. [21] Ann. of Surg., Dec. 1900, p. 749. [22] Med. Rec., New York, Sept. 29, 1900, p. 499. [23] Med. Rec., New York, Jan. 12, 1901 p. 52. [24] Ann. of Surg., Jan. 1901, p. 93. [25] Ann. of Surg. Feb. 1901, p. 173.

